# Pomegranate (*Punica granatum* L.) Attenuates Neuroinflammation Involved in Neurodegenerative Diseases

**DOI:** 10.3390/foods11172570

**Published:** 2022-08-25

**Authors:** Alami Mehdi, Benchagra Lamiae, Boulbaroud Samira, Mhamed Ramchoun, Khalil Abdelouahed, Fulop Tamas, Berrougui Hicham

**Affiliations:** 1 Department of Biology, Polydisciplinary Faculty, University Sultan Moulay Slimane, Beni Mellal 23000, Morocco; 2 Department of Medicine, Geriatrics Service, Faculty of Medicine and Health Sciences, University of Sherbrooke, Sherbrooke, QC J1H 4N4, Canada

**Keywords:** Alzheimer’s disease, *Punica granatum* L., polyphenols, neurodegenerative diseases

## Abstract

Food scientists have studied the many health benefits of polyphenols against pernicious human diseases. Evidence from scientific studies has shown that earlier healthy lifestyle changes, particularly in nutrition patterns, can reduce the burden of age-related diseases. In this context, a large number of plant-derived components belonging to the class of polyphenols have been reported to possess neuroprotective benefits. In this review, we examined studies on the effect of dietary polyphenols, notably from *Punica granatum* L., on neurodegenerative disease, including Alzheimer’s disease, which is symptomatically characterized by impairment of cognitive functions. Clinical trials are in favor of the role of some polyphenols in maintaining neuronal homeostasis and attenuating clinical presentations of the disease. However, discrepancies in study design often bring inconsistent findings on the same component and display differences in their effectiveness due to interindividual variability, bioavailability in the body after administration, molecular structures, cross-blood-brain barrier, and signaling pathways such as nuclear factor kappa B (NF-κB). Based on preclinical and clinical trials, it appears that pomegranate may prove valuable in treating neurodegenerative disorders, including Alzheimer’s disease (AD) and Parkinson’s disease (PD). Therefore, due to the lack of information on human clinical trials, future in-depth studies, focusing on human beings, of several bioactive components of pomegranate’s polyphenols and their synergic effects should be carried out to evaluate their curative treatment.

## 1. Introduction

The improvement of living conditions and access to health care, as well as the progress achieved in the field of public health, have created an increase in the life expectancy of the world’s population. A corollary of this success is the emergence of many age-associated pathologies, such as Alzheimer’s disease (AD). Indeed, the past decades have been marked by a quasi-exponential increase in the prevalence of AD, and the future projections are dramatic. In light of this worrying situation and the absence of curative treatment, the repercussions will exceed the individual medical burden to become considerable social and economic challenges. These considerations, among others, have made AD a major public health problem and a worldwide medical preoccupation.

**Figure 1:**
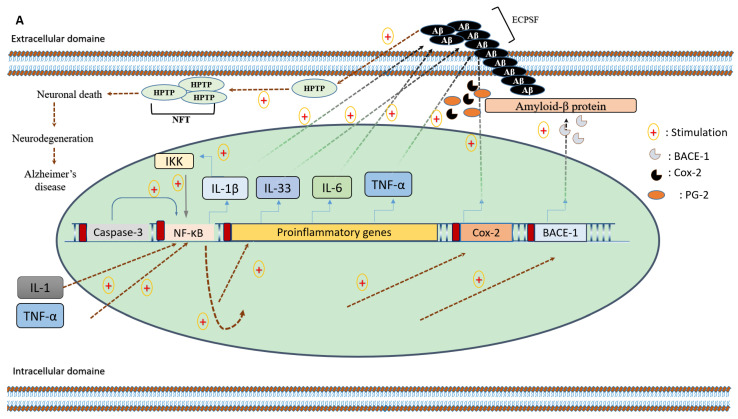

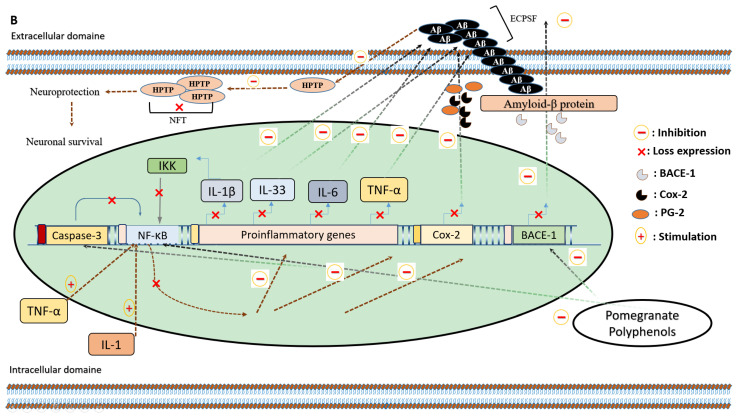
(**A**) The involvement of neuro-inflammation in Alzheimer’s disease. (**B**) The proposed operative anti-neuroinflammatory mechanism and amyloidogenic inhibitory effects of pomegranate bioactive compounds.

Although the etiology of AD is imperfectly understood, it is believed to be related to several brain abnormalities, including a defect in the phagocytosis of amyloid-beta (Aβ)-peptide by microglia and cytosolic aggregation in the axons/neurons of the hyperphosphorylated cytoskeletal Tau protein, leading, respectively, to senile plaques and neurofibrillary tangle formation. These abnormalities are closely connected and strongly associated with neuronal damages, including disruption of the neuronal communication at the synapses, cytoskeleton dissociation, and ultimately neuronal death (Figure 1). Several pathological circumstances, such as infections, ischemia, and toxic product release, stimulate microglia cells to secrete several proinflammatory cytokines and chemokines, which alter the neuronal homeostasis. Evidence regarding the overexpression of tumor necrosis factor alpha (TNF-α), interleukin-1β (IL-1β), interleukin-6 (IL-6), and transforming growth factor-β (TGF-β) in the brain of affected patients and transgenic animal models for AD [1,2,3], the association of some genetic polymorphisms of these mediators and the disease manifestation [4], in addition to the neuroprotective impact attributed to anti-inflammatory drugs [5], have accumulated to support the involvement of neuroinflammation in the pathogenesis of neurodegenerative diseases.

**Figure 2:**
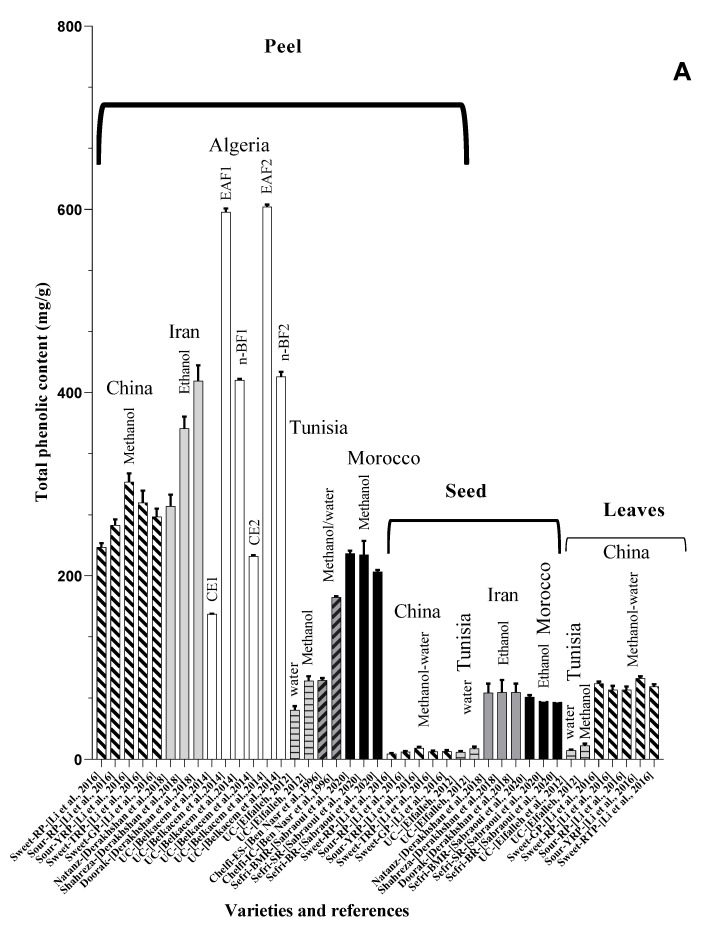

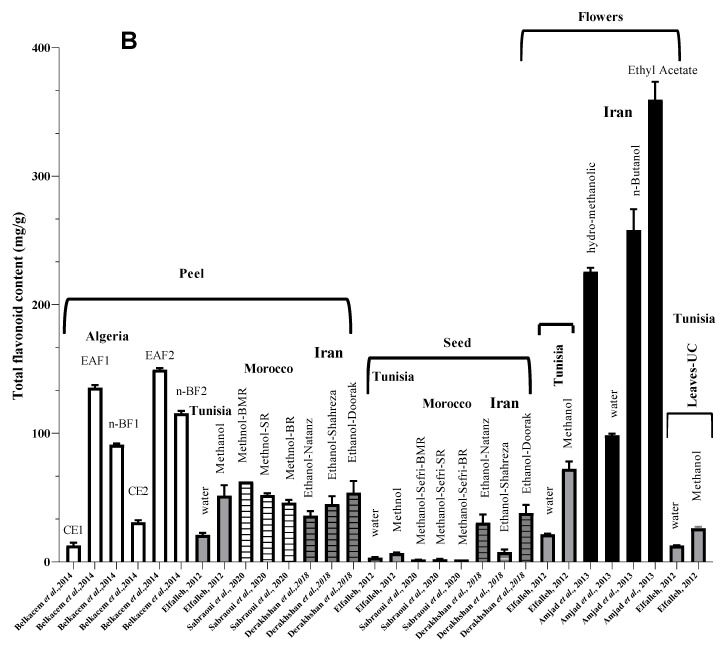
(**A**) Total phenolic content in peels, seeds, and leaves of pomegranate fruit growing in different regions of the world [49,50,52,54,55,56]. BR: Berkane region; SR: Statte region; BMR: Beni Mellal region; UC: unknown cultivar; MWE: methanol/water extract; n-BF1: n-butanol fraction of hydromethanolic extract; CE2: aqueous-acetone crude extract; EAF2: ethyl acetate fraction of aqueous-acetone extract; n-BF2: n-butanol fraction of aqueous-acetone extract; Chelfi-ES: chelfi exposed to sun; Chelfi IC: Chelfi in shade. (**B**) Total flavonoid content in pomegranate leaves, peels, seeds, and flowers, from different geographical areas of the world [49,52,53,54,56]. BMR: Beni-mellal region; SR: Statte region; BR: Berkane region; UC: unknown cultivar. As shown, the Iranian and Algerian varieties seem to accumulate very important quantities of these bioactive constituents. Furthermore, the final results are sensitive to different factors such as the solvent used for the extraction, genetic variabilities, variety, and the geoedaphic and climatic conditions that characterize each country.

Pomegranate, a rich source of secondary metabolites and natural compounds (Figure 2), has emerged as a complementary candidate to synthetic anti-inflammatory agents, and has already proved its effectiveness in the context of various chronic inflammatory diseases [6,7,8,9]. In the neuronal micro-environment, pomegranate polyphenols improve brain neurochemistry through their abilities to inhibit NF-κB actions [6], a redox-sensitive transcription factor that is strongly involved in the mRNA transcription of many pro-inflammatory and toxic biomolecules [7]. Additionally, pomegranate bioeffects also include the reduction of beta-site amyloid precursor protein (APP) cleaving enzyme 1 (BACE1) gene expression [6], cyclooxygenase 2 (Cox-2) enzymatic activities [8], and the catalytic activities of caspase enzymes [9]. These effects are thought to reduce neuroinflammation, restore, or at least maintain neuronal homeostasis, and attenuate clinical presentations of the disease.

This review describes the effects of the metabolite compounds of *Punica granatum* L on the attenuation of neurodegenerative diseases including AD and Parkinson’s disease (PD). Most results discuss the anti-inflammatory component of pomegranate in AD.

## 2. Microglia and Neuroinflammation in the Context of Neurodegenerative Diseases

Neurodegenerative diseases including AD, PD, and amyotrophic lateral sclerosis (ALS) are symptomatically characterized by the impairment of cognitive and/or motor functions. Neurodegeneration in these disorders affects neuronal death, which has been linked to the presence of toxic protein deposits in the central nervous system (CNS), including Aβ and Tau for AD, α-synuclein for PD, and superoxide dismutase 1 (SOD1) and TAR-DNA-binding protein (TDP-43) for ALS [10,11]. Interestingly, these events occur as a result of various neuroinflammatory processes involving glia–neuron cross-talk alterations. Although the genetic and environmental factors that initiate degeneration differ among these diseases, a shared biochemical cascade of inflammatory events plays a central role in mediating neuronal cell loss [12,13,14,15], though it has been well documented that neuroinflammation, largely mediated by microglia, the resident immune cells of the brain, contributes to the onset and progression of neurodegenerative diseases [16].

In a non-pathological context, microglia cells act as macrophages, participate in brain homeostasis, and accomplish several reparative and restorative neuronal functions. This includes the clearance of dendritic debris, synaptic organization, biological response to biotoxins, mediation of inflammation, and the phagocytosis of aggregated proteins [17]. However, the chronic activation of microglia, specifically mediated by microglia proinflammatory M1 phenotype, leads to the production of proinflammatory cytokines that contribute to the progression of various neurodegenerative diseases [18]. The microglia is able to release several signaling molecules required for the maintenance of brain homeostasis through many signaling pathways. Classical NF-κB signaling–NF-κB activation can prevent apoptosis of the cell in which it is activated; it may indirectly lead to apoptosis of other cells by promoting the production of cytotoxic agents and is a major regulator of inflammation, driving the gene expression of pro-inflammatory cytokines, including TNF-α, Il-6, and Il-1β [19], and enzymes such as cyclooxygenase-2 [20] and BACE1 gene expression [21], which have detrimental effects in neurodegenerative diseases (Figure 1A).

In AD patients, morphological changes of microglia surrounding the senile plaques can indicate the level of neuroinflammatory response, the phosphorylation of Tau increases, and the Tau and Aβ pathology exacerbation [22].

Similarly, in Parkinson disease (PD), the over-expression of α-synuclein released from dying dopaminergic neurons drives microglia into a reactive pro-inflammatory phenotype, altering the neuroinflammatory process and promoting neurodegeneration [23,24].

The infiltration of activated microglia observed in ALS patients is responsible for the toxicity caused by mutated SOD1 expression and increases of proinflammatory-activated microglia phenotypes [25].

The same suspects are also established for Huntington’s pathology, to incriminate this exaggerated innate immune response in the necrotic process of neurons and the progressive decline of brain vital capacities [26,27].

In summary, balancing and switching between the phenotypes of microglia at specific times and in specific patients may be important for modulating the progression of neurodegenerative diseases. The accumulation of Aβ and Tau protein, the superoxide dismutase 1 (SOD1) mutant microglia, and the aggregated α synuclein activate microglia through the release of neuroinflammatory mediators that promote neurodegeneration.

Many factors, including microglia phagocytosis incapacities, extracellular misfolded Aβ aggregation, IL-1, and TNF-α cytokines have been proposed to stimulate the NF-κB [28,29]. The activated form of this nuclear factor plays a crucial role in the neuro-inflammatory events that characterizes AD. ECSPF: extracellular senile plaques formation; HPTP: hyperphosphorylated Tau protein; PG-2: prostaglandin-2; IKK: inhibitor of the nuclear factor-κB kinase. Pomegranate polyphenols are thought to be effective in the modulation of the proinflammatory M1 phenotype of microglia. This is probably achieved through its abilities to inhibit NF-κB actions [6], BACE1 transcription [6], and Cox-2 enzymatic activities [8], and to reduce the catalytic activities of the caspase 3 enzyme [9].

## 3. Polyphenols in Clinical Research of Alzheimer’s Disease

Despite the scientific support of the preclinical results of the anti-neuroinflammatory effects of pomegranate bio-nutrients and products, clinical data are not yet available. However, several extracts and biopolyphenols from other natural resources, and the plant kingdom, have been sufficiently tested, in both preclinical and clinical conditions. Indeed, promising achievements in terms of enhancing neurological functions regarding the neuroprotectivity and antisenescence potential of many phytochemical ingredients from natural resources have attracted the attention of the neuroscientific community. In this regard, a placebo-controlled trial performed with melissa officinalis extract has suggested episodic memory and cognitive capacity enhancement in patients with mild to moderate AD [30]. Moreover, AD-related behavioral symptoms were attenuated in a group of patients treated with turmeric drugs, a traditional Indian medicine [31]. Furthermore, eight months of regular ingestion (200 mL/day) of a beverage rich in antioxidant constituents and polyphenols was seen to reduce homocysteine blood levels, a toxic amino acid that exerts direct neurotoxicity and influences AD pathophysiology and pathogenesis [32]. In the same way, a prospective, randomized, double-blind, placebo-controlled study, performed by Choudhary et al. [33], attributed promising efficacy of *Withania somnifera* L. Dunal root extracts against age-associated memory decline in Indian volunteers. Additionally, another prospective study carried out using Greek extra virgin olive oil demonstrated the effectiveness of this treatment in neutralizing cognitive impairments [34].

These clinical investigations attribute a significant positive brain impact to pomegranate consumption. However, some studies present limitations, particularly in terms of study size, treatment duration, and negligence of nutrigenomics variabilities, in addition to the possible influences of hormonal fluctuations, as some trials included women participants.

Pure alkaloids with nitrogen groups and organic biomolecules with hydroxyl radicals may also exert neuroprotective effects regarding their central and peripheric biological activities. The healthy benefits of these bio-agents appear to involve multiple mechanisms and to act at different cellular and molecular locations (oxidant damages attenuation, interference with neuronal signaling pathways, toxic Aβ-deposits reduction, and mediation of parasympathetic activities). In this regard, Huperzine-A, a Chinese Huperzia Serrata naturally-occurring nutrient, with reversible, selective, and linear competitive acetylcholinesterase inhibitory activities, was effective in reducing task switching deficit and cognitive abnormalities in patients with Alzheimer’s pathology [35]. This evidence was supported by Chinese clinical research outcomes, which significantly suggest an enhancement of general cognitive capacities and a remarkable improvement of memory in elderly people suffering from the same disease [36]. Furthermore, Huperzine-A polyphenol interacts with AD-related pathophysiology to reduce clinical symptoms and mitigate memory loss in elderly subjects suffering from neurological diseases [37].

Other non-flavonoid compounds, such as resveratrol micronutrient, a natural food ingredient with higher bioavailability and brain blood barrier penetrability [38], were also investigated for their anti-neurodegeneration potential. In fact, a recent retrospective study attributed an anti-AD efficacy to the resveratrol compound, based on its ability to reduce cerebrospinal fluid matrix peptidases (such as metalloproteinase 9), neuroinflammation, and adaptative immune response [39]. However, opposite findings with resveratrol have been observed, suggesting unexpected results, manifesting in a greater reduction of cerebrospinal fluid and serum Aβ40 concentrations in the placebo group compared to the treated group [38]. In most clinical trials, the psychological effect of the placebo “the placebo effect” is neglected, but it could influence psychology and consequently impacts physiology to obtain such effects.

Even though the positive impact of dietary polyphenols on AD is noticeable, there is a clear gap provided from different results, which are conflicting for many reasons: the routes of administration, bioavailability in the body after administration, molecular structure and properties of polyphenols, the treatment duration, the size of trials and subjects, the study characteristics, and the pathways involvement. However, the new therapeutic approach should be exploited carefully for treating neurodegenerative disease. Ongoing study on polyphenol consumption in patients at risk for AD is significant, but it should be validated and transferred to humans without any risks.

## 4. Nutritional Characterization and Metabolites Compounds of *Punica granatum* L.

Secondary metabolites, such as flavonoids and ellagitannins, are synthesized through the shikimic pathway and have been, for some time, considered as non-necessary metabolites. It was required to wait for the development of a new understanding in the phytochemistry field to highlight their critical involvement in plant development and survival.

Chromatographic analysis of different varieties of pomegranate from different regions of the world shows both quantitative and qualitative diversity in its bioactive compounds (Table 1 and Table 2), with an abundance of the major polyphenolic classes, including, flavonoids (Figure 2B), tannins, and anthocyanin pigments.

**Table 1:** Summary of the most important groups and subgroups of polyphenol compounds present in *Punica granatum* L.

Anthocyanins		
[40]	[40]	[40]
Pelargonidin 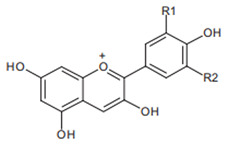 R1 = R2 = H	Cyanidin 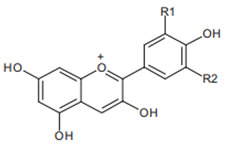 R1 = H, R2 = OH	Delphinidin 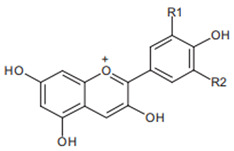 R1 = R2 = OH
**Ellagitannins**		
[41]	[42]	[42]
Punicalin 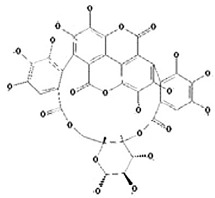	Ellagic acid 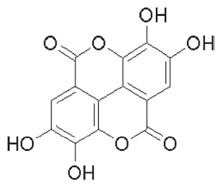	Punicalagin 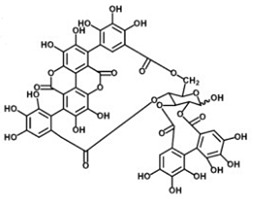
**Flavonoids**		
[41]	[41]	[41]
Luteolin (flavones) 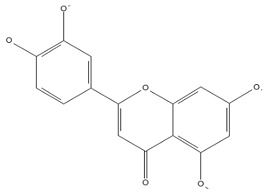	Kaempferol (flavonols) 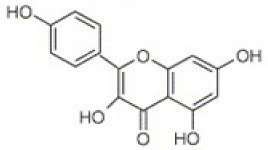	Catechin (flavan-3-ols) 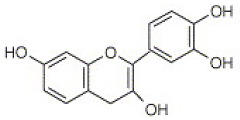

**Table 2:** Summary of quantification findings in terms of total phenolic, total flavonoid, total anthocyanin, and total tannin content in different pomegranate parts and products.

PG Part or Product	Solvent	Variety	Country	TPC	TAC	TFC	TPA	TT	Ref
Data is presented in mg/L									
Juice	Aqueous	Malase Ashkzar	Iran	8130 ± 0.1	654,830 ± 0.1	ni	ni	ni	[43]
		Sweet Aalak		2380 ± 0.0	815 ± 0.0	ni	ni	ni	
		Sooleghan		7440 ± 0.1	5980 ± 0.1	ni	ni	ni	
		Malase Ardestan		7920 ± 0.1	6430 ± 0.2	ni	ni	ni	
		Saveh Black Leather		4200 ± 0.2	2750 ± 0.1	ni	ni	ni	
		Ardestan Black Leather		5820 ± 0.1	4330 ± 0.0	ni	ni	ni	
		Saveh Sweet White Leather		5490 ± 0.2	4070 ± 0.2	ni	ni	ni	
		Ostokhani Tabas		9300 ± 0.1	7760 ± 0.1	ni	ni	ni	
Juice	Aqueous	RPS		1018 ± 22.1	0.87 ± 0.1	104.2 ± 12.5	ni	3.05 ± 0.34	[44]
		RPGH		1082 ± 12.8	0.18 ± 0.04	259.6 ± 9	ni	2.22 ± 0.9	
		ZZ		960 ± 17. 5	0.44 ± 0.1	282.1 ± 11.0	ni	6.33 ± 0.1	
		SZ		1026 ± 21.3	8.22 ± 1.9	342 ± 12.1	ni	4.12 ± 0.8	
		MY		706 ± 23.9	0.63 ± 0.2	320.6 ± 9.9	ni	5.21 ± 1.1	
		MS		1062 ± 14.1	0.48 ± 0.1	14.3 ± 4.8	ni	6.55 ± 0.5	
		B		896 ± 12.1	1.13 ± 0.2	100 ± 10.1	ni	1.01 ± 0.4	
Skin	Methanol	RPS		785 ± 11.1	6.40 ± 1.3	749.5 ± 15.8	ni	3.23 ± 0.2	
		RPGH		645 ± 31.1	0.26 ± 0.03	328.2 ± 13.9	ni	4.46 ± 1	
		ZZ		671 ± 20.02	1.57 ± 0.5	587.2 ± 10.2	ni	1.09 ± 0.7	
		SZ		705 ± 23.3	1.69 ± 0.6	930 ± 16	ni	2.43 ± 1	
		MY		553 ± 12.5	0.05 ± 0.02	502.4 ± 9.1	ni	4.43 ± 2.4	
		MS		597 ± 18.8	0.16 ± 0.01	382.1 ± 17.5	ni	4.22 ± 2.2	
		B		783 ± 10.02	11.20 ± 2.4	712.7 ± 9	ni	4.1 ± 0.8	
Juice	Aqueous	unknown cultivar	Germany	2015.2 ± 21.6	198.3 ± 0.8	ni	6.9 ± 0.0 ^a^ 21.4 ± 0.9 ^b^	423.8 ± 15.3	[45]
				5186.0 ± 172.5	124.2 ± 2.2	ni	1.1 ± 0.0 ^a^ b: nd	2074.4 ± 47.3	
				2122.0 ± 0.0	557.7 ± 48.3	ni	10.6 ± 0.3 ^a^ 28.3 ± 1.4 ^b^	93.2 ± 3.6	
Juice	Aqueous	Ermioni variety	Greece	1271 ± 40	382.8 ± 0.1	ni	ni	ni	[46]
Data is presented in mg/kg									
Peel	Ethanol, formic acid, MeOH, water, and acetonitrile	Italian cultivar	Italy	-	not detected	16,156.4 ± 7450.9	5285.6 ± 9316.1	ni	[47]
Pulp				-	264.3 ± 184.7	344.4 ± 281.4	77.4 ± 33.3	ni	
Peel	Aqueous	unknown cultivar	Germany	44,261.5 ± 414.0	447.1 ± 11.3	ni	270.4 ± 18.5 ^a^ b: nd	43,991.2 ± 395.5 ^c^	[45]
Mesocarp				40,625.1 ± 4434.7	nd	ni	a: nd b: nd	40,625.1 ± 4434.7 ^c^	
Data is presented in mg/g									
Peel	Ethanol, formic acid, methanol, water, and acetonitrile	Gaeta 1	Italy	179.92 ± 1.31	-	-	-	ni	[47]
		Gaeta 3		244.61 ± 1.41	-	-	-	ni	
		Gaeta 4		182.15 ± 1.57	-	-	-	ni	
		Tordimonte A		89.68 ± 0.61	-	-	-	ni	
		Itri A		141.14 ± 0.45	-	-	-	ni	
		Wonderful		137.28 ± 1.19	-	-	-	ni	
		Formia		191.59 ± 3.38	-	-	-	ni	
Pulp		Gaeta 1		8.89 ± 0.08	-	-	-	ni	
		Gaeta 3		5.40 ± 0.09	-	-	-	ni	
		Gaeta 4		4.95 ± 0.06	-	-	-	ni	
		Tordimonte A		3.19 ± 0.07	-	-	-	ni	
		Itri A		6.11 ± 0.07	-	-	-	ni	
		Wonderful		6.14 ± 0.03	-	-	-	ni	
		Formia		5.32 ± 0.05	-	-	-	ni	
Aril	Aqueous	Unknown cultivar		0.040 ± 0.0072	ni	ni	ni	ni	[48]
	Ethyl acetate			0.007 ± 0.0012	ni	ni	ni	ni	
Juice	Aqueous			0.052 ± 0.0001	ni	ni	ni	ni	
	Ethyl acetate			0.001 ± 0.0003	ni	ni	ni	ni	
Rind	Aqueous			0.907 ± 0.0757	ni	ni	ni	ni	
	Ethyl acetate			0.031 ± 0.0003	ni	ni	ni	ni	
Seed	aqueous	Gabsi variety	Tunisia	7.94 ± 1.25	19.62 ± 3.12	3.30 ± 0.52	ni	32.86 ± 4.24 ^c^	[49]
Leave				9.85 ± 0.82	40.91 ± 3.43	12.77 ± 0.23	ni	64.40 ± 4.85 ^c^	
Flower				42.70 ± 2.17	80.20 ± 7.02	21.45 ± 0.58	ni	57.04 ± 3.41 ^c^	
Peel				53.65 ± 4.13	51.02 ± 10.33	21.03 ± 1.62	ni	62.71 ± 11.32 ^c^	
Seed	Methanol			11.84 ± 1.92	40.84 ± 7.77 ^c^	6.79 ± 0.57	ni	29.57 ± 4.54 ^c^	
Leave				14.78 ± 2.10	89.81 ± 7.50	26.08 ± 1.24	ni	128.02 ± 4.49 ^c^	
Flower				66.29 ± 3.06	168.91 ± 3.1	72.52 ± 5.59	ni	148.24 ± 10.29 ^c^	
Peel				85.60 ± 4.87	102.20 ± 16.42	51.52 ± 8.14	ni	139.63 ± 4.25 ^c^	
Peel	Aqueous	Chelfi variety		216.9 ± 7.3	ni	ni	ni	ni	[50]
Fruit	Methanol-water	Unknown cultivar	Algeria	15.39 ± 0.08	ni	12.95 ± 0.07	ni	ni	[51]
Seed	Methanol	Sefri	Morocco (BMR)	67. 85 ± 1.98	ni	1.76 ± 0.02	ni	ni	[52]
			Morocco (SR)	63.34 ± 0.7	ni	2.11 ± 0.28	ni	ni	
			Morocco (BR)	62.17 ± 3.26	ni	1.94 ± 0.00	ni	ni	
Peel			Morocco (BMR)	224.39 ± 3	ni	62.63 ± 3.23	ni	ni	
			Morocco (SR)	223.21 ± 15	ni	52.12 ± 1.36	ni	ni	
			Morocco (BR)	204.58 ± 1.96	ni	46.17 ± 2.18	ni	ni	
Flowers	Hydromethanolic	Unknown cultivar	Iran	348.81 ± 5.58	ni	225.776 ± 2.93	ni	ni	[53]
	Aqueous			509.83 ± 11.61	ni	98.399 ± 1.15	ni	ni	
	n-Butanol			866.47 ± 26.61	ni	258.127 ± 16.19	ni	ni	
	Ethyl acetate			944.75 ± 8.27	ni	359.6 ± 13.91	ni	ni	
Peel	Ethanol	Natanz		276 ± 12.69	ni	36 ±3.56	ni	ni	[54]
		Shahreza		361 ± 12.87	ni	45 ± 6.25	ni	ni	
		Doorak		413 ± 16.84	ni	54 ± 8.96	ni	ni	
Seed		Natanz		72.4 ± 10.02	ni	30.5 ± 6.38	ni	ni	
		Shahreza		73 ± 13.35	ni	7.55 ± 2.12	ni	ni	
		Doorak		73 ± 9.45	ni	38 ± 6.38	ni	ni	
Juice	Ethanol	Natanz		23.8 ± 6.74	ni	1.8 ± 1.03	ni	ni	
		Shahreza		15.8 ± 5.81	ni	2.14 ± 0.92	ni	ni	
		Doorak		12.4 ± 5.21	ni	8.7 ± 2.47	ni	ni	
Peel	Methanol-water	Sweet-GP	China	264.58 ± 8.74	ni	ni	ni	ni	[55]
Flesh				217.14 ± 16.80	ni	ni	ni	ni	
Seeds				9.04 ± 1.23	ni	ni	ni	ni	
Juices				8.62 ± 1.05	ni	ni	ni	ni	
Leaves				82.31 ± 2.26	ni	ni	ni	ni	
Peel		Sweet-RP (Chinese cultivar)		231.36 ± 4.40	ni	ni	ni	ni	
Flesh				203.20 ± 12.68	ni	ni	ni	ni	
Seeds				6.17 ± 0.60	ni	ni	ni	ni	
Juices				6.12 ± 0.91	ni	ni	ni	ni	
Leaves				75.56 ± 4.39	ni	ni	ni	ni	
Peel		Sour-RP (Chinese cultivar)		255.31 ± 6.42	ni	ni	ni	ni	
Flesh				208.39 ± 6.31	ni	ni	ni	ni	
Seeds				8.31 ± 0.83	ni	ni	ni	ni	
Juices				10.36 ± 1.12	ni	ni	ni	ni	
Leaves				75.61 ± 3.62	ni	ni	ni	ni	
Peel		Sour-YRP		302.43 ± 9.54	ni	ni	ni	ni	
Flesh				272.10 ± 9.98	ni	ni	ni	ni	
Seeds				12.44 ± 0.96	ni	ni	ni	ni	
Juices				13.31 ± 0.87	ni	ni	ni	ni	
Leaves				88.33 ± 1.92	ni	ni	ni	ni	
Peel		Sweet-TRP		279.76 ± 13.32	ni	ni	ni	ni	
Flesh				222.62 ± 7.72	ni	ni	ni	ni	
Seeds				8.59 ± 1.04	ni	ni	ni	ni	
Juices				12.91 ± 0.87	ni	ni	ni	ni	
Leaves				79.10 ± 2.74	ni	ni	ni	ni	
Peel	CE1	Unknown cultivar	Algeria	158.18 ± 0.66	ni	12.8 ± 2.2	ni	-	[56]
	CE2			221.54 ± 1.08	ni	30.9 ± 1.6	ni	-	
	EAF1			597.08 ± 3.9	ni	135.5 ± 2.0	ni	-	
	n-BF1			413.6 ± 1.48	ni	91.1 ± 0.88	ni	-	
	EAF2			602.8 ± 2.41	ni	149.4 ± 1.27	ni	-	
	n-BF2			417.56 ± 5.3	ni	115.56 ± 1.81	ni	-	
Data is presented in µg/mg									
Fruit	Water	Unknown cultivar	India	11.99	ni	ni	ni	ni	[57]
	Ethanol			28.13	ni	ni	ni	ni	
	Acetone			6.44	ni	ni	ni	ni	
	Ether			24.13	ni	ni	ni	ni	
	Ethanol-Ether			24.2	ni	ni	ni	ni	
	Ethanol-water			15.12	ni	ni	ni	ni	
	Ethanol-Ether-water			24.5	ni	ni	ni	ni	
Data is presented in mg/mL									
Juice	Aqueous	Gaeta 1	Italy	1.93 ± 0.05	-	-	-	ni	[47]
		Gaeta 3		1.34 ± 0.07	-	-	-	ni	
		Gaeta 4		0.87 ± 0.03	-	-	-	ni	
		Tordimonte A		1.22 ± 0.03	-	-	-	ni	
		Itri A		1.20 ± 0.05	-	-	-	ni	
		Wonderful		1.58 ± 0.02	-	-	-	ni	
		Formia		1.08 ± 0.06	-	-	-	ni	

Abbreviations: TPC: total phenolic content; TAC: total anthocyanin content; TFC: total flavonoid content; TPA: total phenolic acids; TT: total tannin; ^a^: total hydroxybenzoic acids; ^b^: total hydroxycinnamic acids; ^c^: total hydrolyzable tannins; BR: Berkane region; BMR: Beni Mellal region; SR: Statte region; MeOH: Methanolic solution; PGS E1: Pomegranate seed extract protected from light; PGS E2: Pomegranate seed extract exposed to light; CE1: hydromethanolic crude extract; ni: not investigated; nd: not detected; -: investigated but reported by another unit; EAF1: ethyl acetate fraction of hydromethanolic crude extract; n-BF1: n-butanol fraction of hydromethanolic crude extract; CE2: aqueous-acetone crude extract; EAF2: ethyl acetate fraction of aqueous-acetone crude extract; n-BF2: n-butanol fraction of aqueous-acetone crude extract.

Anthocyanins from pomegranate have been sufficiently investigated and are represented by glucosides of pelargonidin, delphinidin, and cyanidin forms [58,59]. These functional compounds are among the major contributors to the pharmacological properties and color qualities of many vegetables and fruits. Indeed, the blue, red, and orange colors have been ascribed to delphinidin, cyanidin, and pelargonium structures, respectively. Progressively, with the advancement of the stage of maturity, an increase in anthocyanins’ proportions and a decrease in the total phenolic content have been recorded [60]. This is reported to be accompanied by a progressive change in the color properties and the decline of the antioxidant capacities [61]. The reported results for anthocyanins appear to vary from one variety to another, and from region to region (Table 2), suggesting the possible influence of genetic factors and environmental conditions.

Flavonoids are another class of polyphenols and are among the most important secondary metabolites detected in vegetables and fruits. Flavonoids identified in pomegranate include, mainly, catechin [62], epicatechin [62], flavan-3-ol [62], quercetin [63], kaempferol [64], luteolin [64], naringin [65], pelargonidin [65], cyanidin [65], cyanidin 3-O-glucoside [66], cyanidin 3.5-di-O-glucoside [66], delphinidin 3-O-glucoside [66], delphinidin 3.5-di-O-glucoside [66], and punicaflavone [67]. The flavonoid-associated beneficial health effects, such as antioxidant [68], anti-mutagenic [68], and anti-inflammatory properties [69], have conferred an added value to this group, with a great impact on pharmaceutical and medicinal preparations.

Organic acids from pomegranate are mainly represented by citric acid, oxalic acid, shikimic acid, acetic acid, maleic acid, fumaric acid, succinic acid, tartaric acid, and ascorbic acid [70]. Phytochemical analysis of six cultivars growing in Georgia shows citric acid as a predominant organic acid in seed, pull, peel, and leaf (315.7 ± 1.0; 826.7 ± 5.4; 766.9 ± 3.2, and 130.2 ± 1.6 mg/100 g, respectively) [71]. Moreover, in the others twenty-five Iranian cultivars, and thirteen Turkish pomegranate varieties, the highest value of organic acid was also recorded for citric acid (3763.6 ± 144.8 mg/100 g, and 8.96 ± 0.45 g/L, respectively) [70,72]. Other organic acids, including oxalic, succinic, tartaric, and ascorbic acid, were also detected in pomegranate, but at lower amounts [70,72].

The tannin class is extensive, and tannins are the most significant phenolic compounds present in pomegranate. These include, mainly, pomegranatate [73], punicalin [74], 4.4-di-O-methylellagic acid [75], 3-O-methyl-3.4-methylenedioxy [75], 3-O-methylellagic acid [75], punicafolin [76], punicacortein [77], gallic acid [78], ellagic acid [79], and punicalagin [74,80]. In vitro and in vivo study has listed many neuroprotective actions against AD for this last compound [6], and it is suspected to be mediated by its gut microbiota-derived molecules, namely urolithins. This idea is supported by two concerns: the well-documented antioxidant and anti-inflammatory capacities attached to urolithin structures [81], and secondly, to their exclusive ability to penetrate the blood-brain barrier [82].

## 5. Pomegranate Consumption and Neurodegenerative Disorders

### 5.1. Anti-Inflammatory Effects of Pomegranate Intake on Alzheimer’s Disease

One of the first descriptions of the probable involvement of immune mechanisms in AD were made by Rogers et al. in 1988 [83]. As we discussed above, persistent activation of glial cells and astrocytes is suspected of being responsible for multi-neurological illnesses, including AD. This is supported by evidence from genetic investigations, which have linked the genetic polymorphisms of some pro-inflammatory cytokines to the pathological expression of the disease. Furthermore, a meta-analysis of observational studies suggests that chronic utilization of non-steroidal anti-inflammatory drugs could decrease the risk of developing AD [5]. However, most of these drugs are synthetic, expensive, and may present serious side effects. Therefore, it is of paramount importance to envisage and nominate new safe and effective agents that can be used as nutraceuticals and can be easily integrated into a healthy diet, such as the Mediterranean diet.

Bioactive compounds from medicinal plants offer an alternative and have emerged as a key strategy to surmount the mentioned limitations. Pomegranate is one such medicinal plant and contains a considerable number of polyphenols and natural pigments known for their antioxidant and anti-inflammatory properties. This last pharmacological activity of pomegranate has been extensively investigated in the context of several chronic inflammatory diseases [84,85,86,87,88]. Nevertheless, there are limited published investigations [3,6,89] that have focused their research interests on its capacities to attenuate the neuroinflammatory cascade involved in AD.

Evidence from transgenic animal models of AD (APPsw/Tg2576 mice) has proven the significant impact of long-term (15 months) diet supplementation with pomegranate fruit (4% *w*/*w*) on the level of the main pro-inflammatory cytokines, namely IL-2, IL-3, IL-4, IL-5, IL-6, IL-9, IL-10, IL-1β, and TNF-α, with interesting anti-amyloidogenic effects [3] (Figure 1B). Moreover, another study suggests that the same dose and duration of treatment were also effective in reducing the expression of BACE1, soluble amyloid precursor protein-β (sAPP-β), insulin-like growth factor-1 (IGF-1), inducible nitric oxide synthase (iNOS), C-terminal fragment-β (CTF-β), brain-derived neurotrophic factor (BDNF), postsynaptic density proteins-95 (PSD-95), and phosphorylation of calcium/calmodulin-dependent protein kinase II-α (p-CaMKII-α/CaMKII-α) [90]. Furthermore, Kim et al. [6] tested the therapeutical capacities of punicalagin, an ellagitannin-derived compound, on neuroinflammation and memory impairment. The research outcomes suggest that this compound may reduce Aβ 1-42, IL-1β, IL-6, and TNF-α levels, and neuronal expression of BACE1. Its capacities to attenuate NF-κB DNA binding activity and to inhibit the nuclear factor of kappa-B inhibitor alpha (IkB-α) phosphorylation were also reported by the same authors. To this anti-inflammatory potential, antioxidant activities have also been mentioned, and are exemplified mainly in the free radical-scavenging activities and the protection of brain lipids from the peroxidation cascade. However, all the animal models used in these studies present limitations. This usually manifests in their inability to reproduce the broad spectrum of the disease (low “face validity”) as occurring in humans, as well as in the structural and biochemical non-similarity of amyloid deposits between animal models of AD and affected human subjects [91].

Most of the in vivo-observed effects were also well-established in an in vitro context (Table 3). Pomegranate juice (25–200 μg/mL) treatment of human neuroblastoma cells (SK-N-SH), was correlated with the reduction of cyclooxygenase 2 expression, and consequently with prostaglandin E2 production [8]. An inhibitory effect of IκB, IKK, and BACE1 gene expression and Aβ peptide production were also registered. The same effects on cyclooxygenase 2 and BACE1 expressions were obtained in a co-culture of primary astrocytes and BV-2 cells cultivated with the punicalagin compound (10, 20, and 50 µM) [6]. Additionally, an inhibitory action is also observed against phosphorylation of IkB-α. This last effect is of fundamental importance to the inflammatory pathway, both on the capacity of IkB-α to govern NF-κB pathways and on related activities. The results from Rackova et al. [92] strengthen this evidence and attribute an important neuroprotective potential of pomegranate against glial cell-mediated neuroinflammation.

The neuroprotective ability of pomegranate and its abilities to improve, or at least to maintain, cognitive performances have been scarcely investigated in human subjects. Until now, and to the best of our knowledge, there is only one clinical trial [93] that has investigated the effects of long-term pomegranate juice supplementation on healthy middle-aged and older adults. In this randomized placebo-controlled research, the pomegranate molecules were effective in stabilizing visual memory capacities. However, no other conclusive effects were observed in these healthy subjects.

The discussed benevolent effects are thought to be associated with its capacity to inhibit the cyclooxygenase pathway and the enzymatic activities of β and γ secretase [8]. The inflammatory lowering effect is well documented as being related to the modulation of the NFκB signaling pathways (Figure 1B) [6,8]. Other mechanisms, such as radical scavenging properties, and the organism’s antioxidant status improvement may also contribute to the observed neuroprotective impact. Human studies are badly needed to corroborate the in vitro and animal studies.

### 5.2. Anti-Inflammatory Effects of Pomegranate Intake against Parkinson’s Disease

To date, there are few studies that have investigated the possible link between pomegranate intake and neuro-inflammation implicated in PD [95,96]. The short-term (two weeks) diet supplementation with pomegranate juice (6.5–7.5 mL/day) of male Lewis rats was found to be associated with the aggravation of the inflammatory response, dopaminergic neuronal death, and caspase activity improvement, with no effect on postural instability [95]. These unexpected results have attracted more attention, regarding the anti-inflammatory and antioxidant activities attached to pomegranate consumption [97,98,99]. Numerous factors have been proposed to explain these interesting findings, among them the fact that polyphenol constituents may act as a pro-oxidant compound with all known associated deleterious consequences on neuronal components. Five years later, a paper appeared to suggest opposing results [100]. In this research, the anti-Parkinson’s disease effects are attributed to pomegranate consumption (500 mg/kg/day), and evidenced by anti-α-synuclein aggregation, oxidative damage attenuation, neuronal survival improvement, and postural stability enhancement. Similar findings have been obtained by [96] in a rat model of Parkinson’s disease, using standardized pomegranate extract (40% ellagic acid). The pomegranate-treated groups exhibit a reduction in malondialdehyde, ILβ-1, and TNF-α levels, in addition to the reduction of iNOS activity and apoptotic caspase-3 expression. Furthermore, this phenolic extract also increases cellular brain viability and locomotor performances. However, all these studies have used Rotenone to induce Parkinson’s disease-like-pathology, and they are not based on the same dose, duration of treatment, and animal model (Table 4). Additionally, the artificial method used for the disease induction does not simulate the normal pathological process. Consequently, this may impact the results and contribute to explaining, at least in part, the contradictory reported outcomes.

### 5.3. The Preventive Role of Pomegranate against Multiple Sclerosis

Limited research has focused on the ability of pomegranate polyphenols to prevent the expression and development of multiple sclerosis abnormalities. Scopus, web of science, and PubMed database research show the existence of only one paper that has concentrated its focus of interest on pomegranate intake and neuro-inflammation implicated in multiple sclerosis [101]. In this publication, pomegranate peel extract was effective in reducing the level of Il-17. This may interfere with multiple sclerosis expression besides its ability to attenuate the activation of encephalitogenic T-cells. Such an event is fundamental in multiple sclerosis and encephalomyelitis disease development [102]. Moreover, pomegranate seed oil has been proven to inhibit demyelination, a seminal feature of the disease, in addition to its great capacities to attenuate brain lipid peroxidation [103]. However, it is very difficult to generalize these outcomes due to the limited amount of research, and it will be essential to await future studies for probable confirmation or information of the mentioned effects. Therefore, we have highlighted the need to perform more research to further explore and to clarify the effect of pomegranate intake on this pathology.

## 6. Conclusions

We reviewed the roles of polyphenols from pomegranate in neurodegenerative diseases, mostly focusing on AD and PD. In addition, clinical studies on pomegranate treatment associated with the neuroinflammation involved in neurodegenerative diseases were discussed, even though the data was insufficient. From that, it appears that a balance between the neuroprotective glial cells involved in glia-neurons cross-talks may be critical in the progression of neurodegenerative pathologies. Disease stage and complexity, the microglial phenotypes, the polyphenols potents, the routes of administration, and the bioavailability of polyphenols in the brain may influence the neuroprotective benefits of pomegranate’s components against neurodegeneration. Further in-depth studies should be conducted in the future based on human beings and carried out to investigate the curative treatment of pomegranate’s polyphenols, established on the new synergic approaches between different components of pomegranate and neuroinflammation in neurodegenerative disorders.

## Figures and Tables

**Table 3:** Summary of the main in vivo and in vitro findings related to the effect of the pomegranate intake on memory and neuro-inflammation implicated in Alzheimer’s disease.

Animal Model/ Cell Line/CTI	Induction of Neuro-Inflammation	Pomegranate Part or Product	Dose and Period of Treatment	Findings	Ref
Animal studies					
Male C57BI/6 mice	Chronic injection of Aβ peptide	Pomegranate peel extract	800 mg/kg/day for 35 days	Improves spatial memory, and the expression of neurotrophin BDNF; reduce amyloid plaque density, AchE activity, lipid peroxidation and TNF-α level; no hepatic lesions.	[89]
Male ICR mice	LPS injection (250 µg/kg)	Punicalagin	1.5 mg/kg for 4 weeks	Memory improvement; no effect on swimming speed; reduction of BACE1 expression and Aβ 1-42 generation; no effect on APP expression; decrease the Il-1-β, IL-6 and TNF-α, H_2_O_2_ and MDA level; increase GSH/GSSG ratio; inhibition of iNOS and Co-2 activities; decrease NF-kB DNA binding activity; suppress IkB-α phosphorylation and P50 and P65 subunits translocation.	[6]
APPsw/Tg2576 mice	Experimental animal model overexpressing APP	Pomegranate fruit	4% *w*/*w*, for 15 months	Decrease serum concentration of IL-2, IL-3, IL-4, IL-5, IL-9, IL-10, and Eotaxin; decrease the level of IL-1β, TNF-α, and IL-6, Aβ1–42 and 40, both in the cortex and hippocampus areas.	[3]
APPsw/Tg2576 mice	Transgenic female	Pomegranate extract	4% *w*/*w*, for 15 months	Significant inhibition of TNF-α, Il-1-β, iNOS, ccl2, and Il-10 genes expression.	[90]
APP/PS1 transgenic mice	Transgenic female	Urolithin A	300 mg/kg, for 14 days	Neuronal death attenuation; hippocampal neurogenesis improvement; diminished astrocytes and microglia activation; reduction in the level of IL-1β, IL-6, and TNF-α.	[94]
In vitro studies					
The human neuroblastoma (SK-N-SH) cells	IL-1 β (10 U/mL)	Standardized Pomegranate fruit juice (skins)	25–200 μg/mL, for 24 h	Inhibition of Co-2 activity, and PGE-2 production; significant inhibition of IκB and IKK phosphorylation; reduction of BACE1 gene expression and Aβ production.	[8]
HEK293 cells	TNFα (1 ng/mL)		25–200 μg/mL, for 6 h	Significant inhibition of NF-κB transactivation.	
Primary astrocyte and microglial BV-2 cells	LPS-(10, 20 and 50 µM), for 1 h	Punicalagin	(1 µg/mL), for 24h	Suppress the activation of NF-kB; inhibit IkB degradation and p50 and p65 translocation; inhibit APP and BACE1 transcription.	[6]
Microglial BV-2 cell	LPS	Pomegranate seed oil	25 μg/mL, for 24h	Slight inhibition of TNF-α and iNOS gene expression; prevent microglia cells apoptosis.	[92]
Clinical trials					
NCT02093130	_	Pomegranate juice	236.5 mL, for 12 months	Pomegranate juice supplementation stabilizes human capacities in terms of the acquisition of new visual information during advancement with age.	[93]

CTI: clinical trial identifier; BDNF: brain-derived neurotrophic factor; TNF-α: tumor necrosis factor-alpha; Aβ1-42: amyloid beta 1-42; BACE1: beta-site APP cleaving enzyme 1; APP: Amyloid precursor protein; IL-1β: interleukin-1β; IL-6: interleukin-6; H2O2: hydrogen peroxide; MDA: malondialdehyde; GSH: reduced glutathione; GSSG: oxidized glutathione; iNOS: inducible nitric oxide synthase; Co-2: cyclooxygenase 2; AchE: acetylcholinesterase; NF-κB: nuclear factor-kappa B; IkB: inhibitor of nuclear factor-κB; P50: p50 protein; P65: p 65 protein; IL-2: interleukin-2; IL-3: interleukin-3; IL-4: interleukin-4; IL-5: interleukin-5; IL-9: interleukin-9; IL-10: interleukin-10; PGE-2: prostaglandin-2; IKK: inhibitor of nuclear factor-κB kinase; SK-N-SH: human neuroblastoma cell line. BV-2: murine microglial cell line; HEK293: human embryonic kidney cells-293; Ccl2: chemokine ligand 2; LPS: Lipopolysaccharide.

**Table 4:** Summary of the main findings related to Pomegranate supplementation effect on Parkinson’s disease development.

Animal Model	Induction of Parkinson’s Disease	Pomegranate Part or Product	Dose And Period Of Treatment	Findings	Ref
Male Lewis rats	intraperitoneal administration of Rotenone (3 mg/kg)	Pomegranate juice	6.5–7.5 mL/day, for 2 weeks	↑ iNOS expression and activation; ↔ IL-1β; ↔ TNF-α level; ↔ Cox2 expression; ↑ p65 catalytic subunit of the NF-κB; ↑caspase-3 activity.	[95]
Male albino rats	Rotenone (2.5 mg/kg), for 30 days	Pomegranate extract	150 mg/kg/day, for 30 days	↑ locomotor activity; ↓ AchE activities; ↑ BDNF and glutamate; ↓ lipid peroxidation; ↑ SOD; ↑ TAA; ↓ IL-1β; ↓ TNF-α; ↓ iNOS.	[96]
Male albino Wistar rats	subcutaneous injection of Rotenone (1.3 mg/kg), for 35 days	Pomegranate juice	500 mg/kg/day, for 45 days	↑ postural stability; ↑ neuronal survival; ↓ MDA; ↓ α-synuclein; ↑ mitochondrial ALDH activity; ↑ antiapoptotic Bcl-xL protein.	[100]

↑: increase; ↓: decrease; ↔: no effect; iNOS: inducible nitric oxide synthase; IL-1β: interleukin 1-β; TNFα: tumor necrosis factor-alpha; Cox-2: cyclooxygenase 2; NF-κB: nuclear factor-kappa B; AchE: acetylcholinesterase; BDNF: brain-derived neurotrophic factor; TAA: total antioxidant activity; SOD: superoxide dismutase; P65: p 65 protein; MDA: malondialdehyde; ALDH: aldehyde dehydrogenases.

## Data Availability

Not applicable.
